# Tunable-Focus Liquid Lens through Charge Injection

**DOI:** 10.3390/mi11010109

**Published:** 2020-01-20

**Authors:** Shizhi Qian, Wenxiang Shi, Huai Zheng, Zhaohui Liu

**Affiliations:** 1 Department of Mechanical and Aerospace Engineering, Old Dominion University, Norfolk, VA 23529, USA; 2 School of Power and Mechanical Engineering, Wuhan University, Wuhan 430072, China; 2017302650020@whu.edu.cn (W.S.); huai_zheng@whu.edu.cn (H.Z.); 3 School of Energy and Power Engineering, Huazhong University of Science and Technology, Wuhan 430074, China; zliu@mail.hust.edu.cn

**Keywords:** tunable focus, liquid lens, electrohydrodynamics, charge injection

## Abstract

Liquid lenses are the simplest and cheapest optical lenses, and various studies have been conducted to develop tunable-focus liquid lenses. In this study, a simple and easily implemented method for achieving tunable-focus liquid lenses was proposed and experimentally validated. In this method, charges induced by a corona discharge in the air were injected into dielectric liquid, resulting in “electropressure” at the interface between the air and the liquid. Through a 3D-printed U-tube structure, a tunable-focus liquid lens was fabricated and tested. Depending on the voltage, the focus of the liquid lens can be adjusted in large ranges (−∞ to −9 mm and 13.11 mm to ∞). The results will inspire various new liquid-lens applications.

## 1. Introduction

There has been a long history since liquid surfaces were adopted as optical lenses [1,2]. Liquid lenses have many advantages over traditional solid lenses, including simplicity, low cost, smoothness, and flexibility [3,4]. Additionally, liquid lenses can be easily integrated into the optical system or the observed objects [5,6]. As liquid surfaces can be flexibly adjusted and form various geometries, liquid lenses can realize many optical functions, such as in varying the focus [7], optical waveguides [8,9], gratings [10,11], liquid lenses [12,13,14], optical switches [15], optical attenuators [16], and optofluidic prisms [17].

In order to utilize the advantages, it is essential to manipulate the liquid surface morphology. Thus, various efforts have been devoted to controlling liquid surfaces [18,19]. Due to the surface tension effect, liquid surfaces usually present a spherical shape and capillary pressure exists on the surfaces. The capillary pressure varies with the surface morphology [20]. Thus, external forces are introduced to balance the capillary pressure, and the liquid surface morphologies are tuned by the imposed external forces [21]. Hydraulic force is the most common one, actuated by mechanical pumps [22]. However, this technique needs complex liquid transfer systems [23]. A thermal stimulus can be introduced into the manipulation mechanism [24,25]. Thermal actuation can lead to liquid expansion and change the liquid surface [24]. Additionally, thermal actuation can change the surface tension and, consequently, the capillary pressure [25]. However, it is difficult to precisely control liquid surfaces through the thermal method. In addition, for evaporable liquid, the thermal stimulus has some adverse effects. Stimuli-responsive hydrogel can also be employed as a viable tool for manipulating the curvature of the water–oil interface to produce micro lenses with variable focal lengths [26].

Apart from the above methods, the electrically driven method is the most common technique used for manipulating liquid lenses [27,28,29]. The electrically driven mechanism mainly consists of electrowetting [27,28] and dielectrophoresis [29]. Owing to high accuracy and flexibility, the electrically driven method has been widely investigated for many applications, such as adaptive optics, optical switching, and displays. It shows the greatest potential for becoming the dominant technique. However, the current electrically driven methods need complex electrode configurations, which increase the fabrication cost and limit their application.

In this study, we proposed a simple and easily implemented electrically driven method for the liquid lens through charge injection. A prototype was designed and fabricated with a 3D printer, and the proposed method was experimentally validated. The optical performance of the tunable-focus lens by charge injection was characterized and analyzed.

## 2. Principle and Experiments

**Figure 1:**
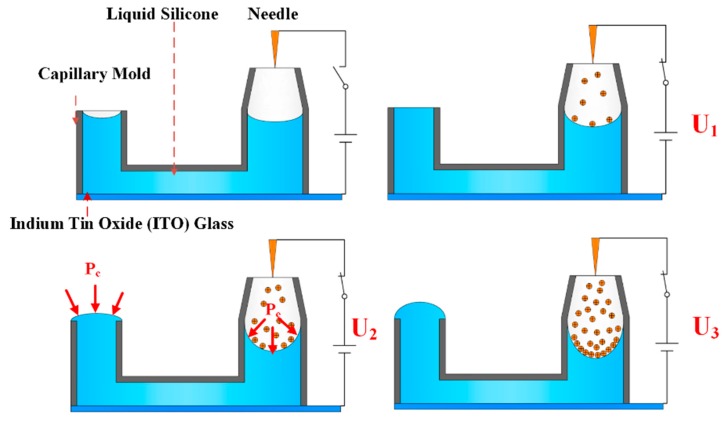
Principle of tunable-focus liquid lens by charge injection. As the imposed voltage increased (i.e., U_3_ > U_2_ > U_1_ > 0), more positive ions accumulated at the air/liquid interface in the right-hand end of the tube, and the induced electropressure pushed the fluid toward the left-hand end of the tube.

Figure 1 schematically depicts the principle of the proposed tunable-focus liquid lens by charge injection. A U-shaped tube with ends of different heights was used to connect the dielectric liquid. A needle-plate electrode configuration was placed in the higher tube end (the right-hand end in Figure 1) and at the bottom of the channel. When a high voltage was applied between the two electrodes, a corona discharge phenomenon occurred. Positive ions generated by the needle drifted toward the plate electrode, were deposited, and accumulated at the interface of the air and the dielectric liquid within the right-hand end of the tube due to the lower electrical conductivity of the liquid. Interactions between the imposed electric field and the ions that had accumulated at the interface generated a Coulomb force, which induced pressure at the interface. We named the induced pressure electropressure, *P_e_*. Under electropressure, liquid flowed to the left-hand, lower tube end, as schematically shown in Figure 1. With an increase in voltage (i.e., U_3_ > U_2_ > U_1_), the electropressure increased. The initial liquid morphology in the left-hand end of the tube had a concave shape, and its capillary pressure, *P_c_*, was negative. The flow created by the induced electropressure pushed the liquid outside and pinned it to the left-hand tube end. Then, the liquid morphology in the left-hand end of the tube became convex in shape and its radius decreased. As the voltage increased, the capillary pressure turned from negative to positive and its value increased to balance the increasing electropressure.

**Figure 2:**
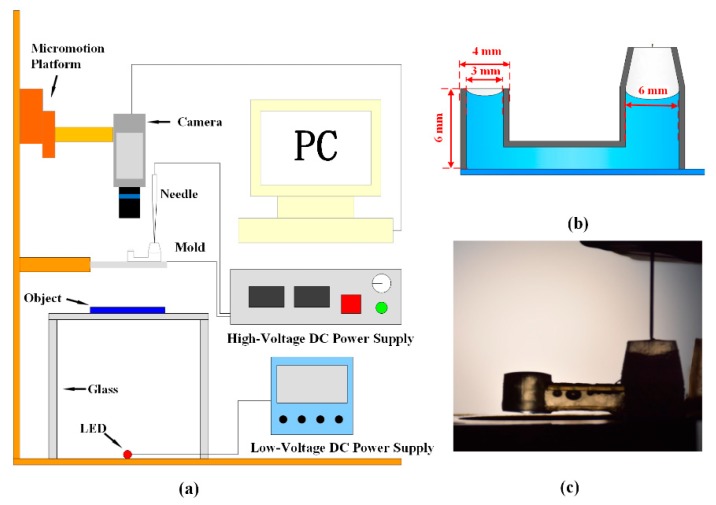
Experimental setup. (**a**) Schematic of experimental setup. (**b**) Schematic of U tube. (**c**) Picture of needle and U tube.

Figure 2a schematically shows our experimental setup. A needle-plate electrode configuration was applied to generate a corona discharge. The curvature radius of the needle electrode made of steel was about 30 µm. The indium tin oxide (ITO) glass plate with a thickness of 2 mm was used as the plate electrode and to support the 3D-printed U-tube and the dielectric liquid. The 3D-printed U-tube was made of a kind of curing polymer using the stereolithography method on ITO glass. The curing polymer was a dielectric material. We used 3D-printing equipment (Form2, Formlabs, Somerville, MA, USA) to print the U-shaped tube. The thickness of the tube wall was 0.5 mm. The left-hand end of the tube had an inner diameter of 3 mm, an outer diameter of 4 mm, and a height of 6 mm, while the other larger end had an inner diameter of 6 mm. A high-voltage direct current (DC) power source (DW-P303-5ACCC, Dongwen Corp., Tianjin, China) connected the two electrodes with the needle as the anode and the ITO plate as the cathode. The voltage could be changed from 0 to 30.0 kV continuously. The distance between the needle electrode tip and the ITO electrode was controlled by a micro motion frame during all the experiments. A digital camera (C13440, Hamamatsu, Japan) was adopted to record the liquid surface and measure the lens focus. When the camera was placed vertically, it was used to record the image of the object; when it was placed horizontally, it took photographs of the liquid morphologies. A LED lamp was used to illuminate the object, and a low-voltage power was applied to light the lamp.

A dielectric fluid, silicone (OE-6650, Dow Corning, Midland, MI, USA), was adopted in all experiments. Its viscosity, electrical conductivity, and surface tension are about 4.0 Pa∙s, 10^−8^ μS/cm, and 0.021 N/m, respectively. Its refractive index is 1.47. The liquid silicone with a volume of 25 μL was first injected using a microliter syringe with a needle into the U-tube, as shown in Figure 2. There were some air bubbles trapped in the U-tube during the injection process. Due to the low density of the bubbles, some of them moved toward the upper ends of the liquid and escaped at the open surfaces in the two ends of the tube. However, some bubbles were trapped near the upper wall of the center part of the U-tube, as shown in Figure 2c. These bubbles, however, did not affect the lens performance, because they remained almost at the same locations and were far away from the light and charge transfer paths. After loading the liquid into the tube, the high-voltage DC power source was turned on and the corona discharge phenomenon occurred.

## 3. Results and Discussion

**Figure 3:**
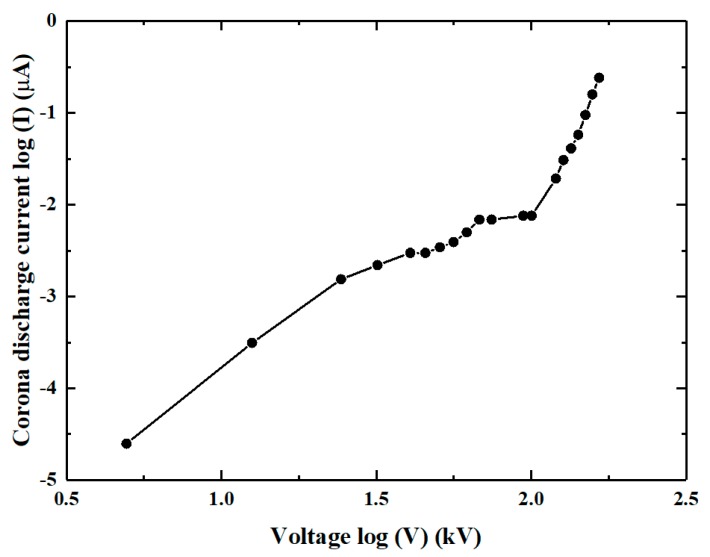
Corona discharge characteristics of the needle-plate electrode configuration.

We first measured the corona discharge characteristics. Figure 3 shows the corona current as a function of the corona voltage imposed between the needle-plate electrodes in the log‒log format. From this figure, we can see that the relationship between the current (I) and the voltage (V) almost follows I α V^n^. In our experiments, different voltages ranging from 0 to 9.2 kV were applied to the two electrodes and the resulting corona currents were measured. The corona discharge occurred at about 2.0 kV with a current of 0.01 µA. The corona current increased slowly with an increasing voltage from 2.0 to 7.0 kV and increased rapidly with an increasing voltage from 7 to 9.2 kV. At a voltage of 9.2 kV, the current reached 0.54 µA. An arcing appeared when the voltage was larger than 9.2 kV. During the whole corona discharge process, power consumption was less than 5 mW.

**Figure 4:**
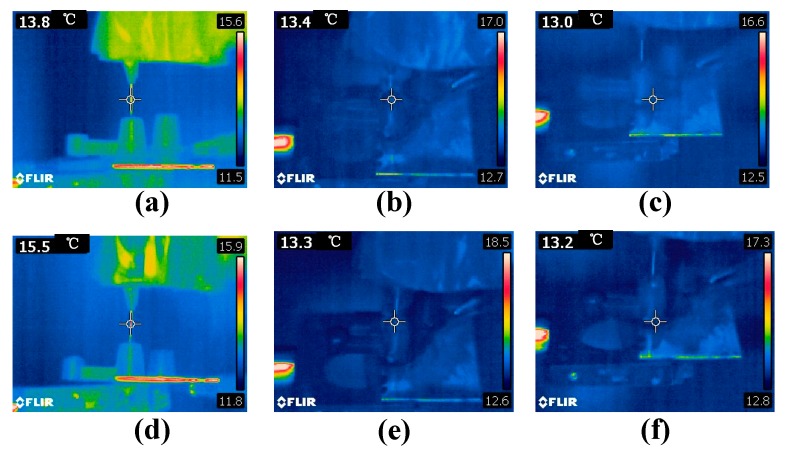
Temperatures of the two electrodes and the liquid silicone at the applied voltages of 2 kV and 9.2 kV: (**a**–**c**) are the infrared images of the needle, liquid, and indium tin oxide (ITO) glass at the voltage of 2.0 kV, while (**d**–**f**) are the infrared images of the needle, liquid, and ITO glass at the voltage of 9.2 kV.

We measured the temperatures of the two electrodes, the needle, the ITO glass, and the liquid silicone using an infrared camera (FLIR, E6, Wilsonville, OR, USA) at a room temperature of 12.5 °C. Figure 4 shows the temperatures at the applied voltages of 2 kV and 9.2 kV, respectively. Within the range of the applied voltages, the temperatures of the electrodes and the liquid silicone did not deviate significantly from the room temperature. The maximum temperature occurred at the needle tip when the applied voltage was 9.2 kV. Thus, the corona discharge did not introduce an obvious thermal effect, due to very low power consumption and heat dissipation to the environment by natural convection.

**Figure 5:**
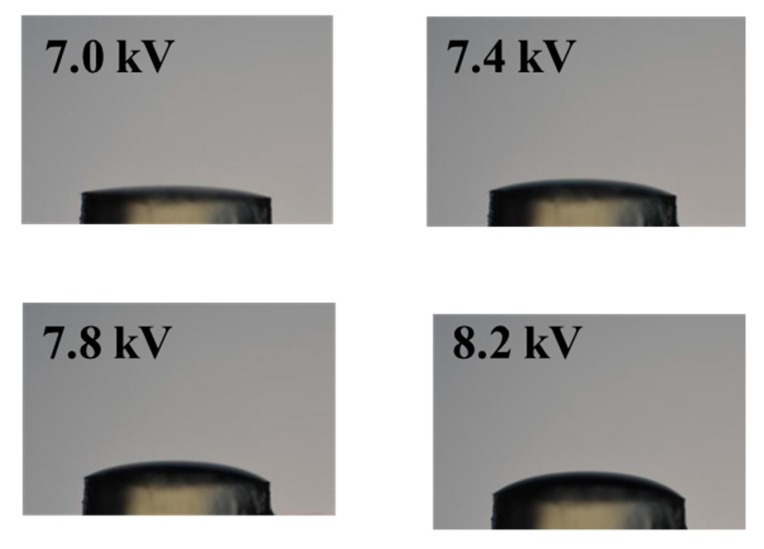
Surface evolution of the liquid lens with an increasing voltage, which shows the liquid silicone bulging outside of the tube.

The side-view images of the liquid lens were recorded by the digital camera. The liquid surface morphologies are shown in Figure 5. Due to the low transparence of the 3D-printed tube, we could not observe the liquid surface inside the tube. Figure 5 only shows the liquid surface morphologies when the liquid was bulged outside the tube. The liquid was pinned at the outer edge of the tube and presented a convex shape. With an increase in voltage, the height of the liquid lens increased. Through imaging processing software, Image J (version 1.8.0, National Institutes of Health, Bethesda, MD, USA), the height could be measured based on the obtained images. For example, when the voltage was 8.2 kV, the height was 0.82 mm.

**Figure 6:**
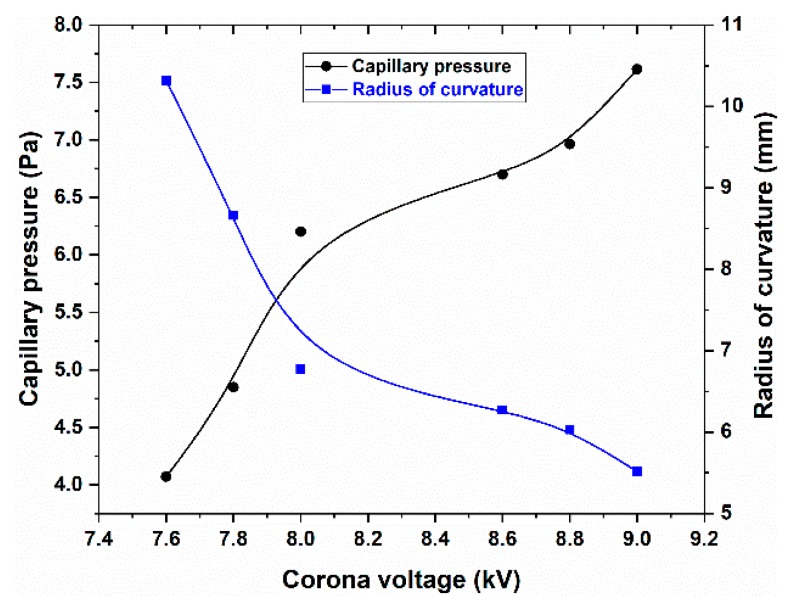
Capillary pressure and curvature radius of the liquid lens as a function of the voltage when the liquid silicone bulged outside of the tube.

The liquid surface morphologies in Figure 5 assumed spherical shapes. The radius of the spherical cap can be calculated by

(1) $$R_{s}=\left(h^{2}+\frac{D^{2}}{4}\right)/2h$$

where *R_s_* is the radius of the spherical cap and *h* is the height of the liquid lens. *D* is the outer diameter of the tube, and its value is 4 mm. The capillary pressure of the liquid lens can be calculated according to Laplace’s law,

(2) $$P_{c}=2\gamma /R_{s},$$

where γ is the surface tension of the liquid silicone with a value of 0.021 N/m. Based on Equations (1) and (2), we can obtain the curvature radiuses and the capillary pressures of the liquid lens at different voltages. The results are shown in Figure 6. The dots are the measured or calculated data. The two solid lines were obtained by fitting the data according to the B-spline algorithm, which shows the dependence of capillary pressures and curvature radiuses on voltages. As the voltage increased from 7.6 to 9.2 kV, the curvature radius decreased from 10.3 to 5.5 mm, while the capillary pressure increased from 4.0 to 7.6 Pa.

**Figure 7:**
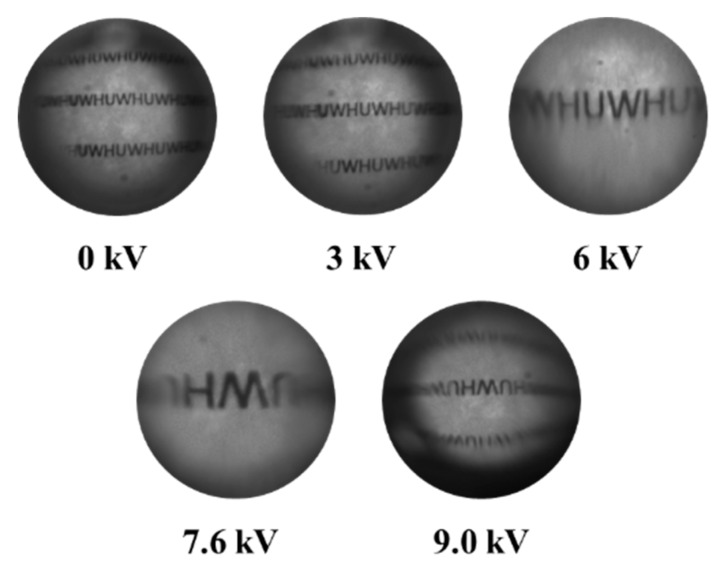
Recorded images of the characters “WHU” through the tunable-focus lens at different voltages.

Figure 7 depicts the images of the characters “WHU” through the tunable-focus lens. The characters were clear in all the images. With an increasing voltage, the images of the characters enlarged. When the voltage was larger than 7.6 kV, the images of the characters were inverted. As the voltage increased further, the characters became smaller. Pincushion distortion was not observed in the images, which suggests that the surface curvature was uniform.

If the liquid surfaces assume a spherical shape, the focal length of the liquid lens can be calculated based on the curvature radius, *R_s_*, as follows:

(3) $$F=R_{s}/\left(n_{l}-1\right)$$

where *F* is the focal length and *n_l_* is the refractive index of the liquid silicone with a value of 1.47.

The focal length of the liquid lens can also be calculated from the images shown in Figure 6. Considering the droplet as a thin lens, *F* was calculated using the following equation:

(4) $$F=h_{i}l/\left(h_{i}+h_{0}\right)$$

where *h_i_* is the image size of the object with the liquid lens, *l* is the distance between the lens and the test object, and *h*_0_ is the image size of the object without the liquid lens.

**Figure 8:**
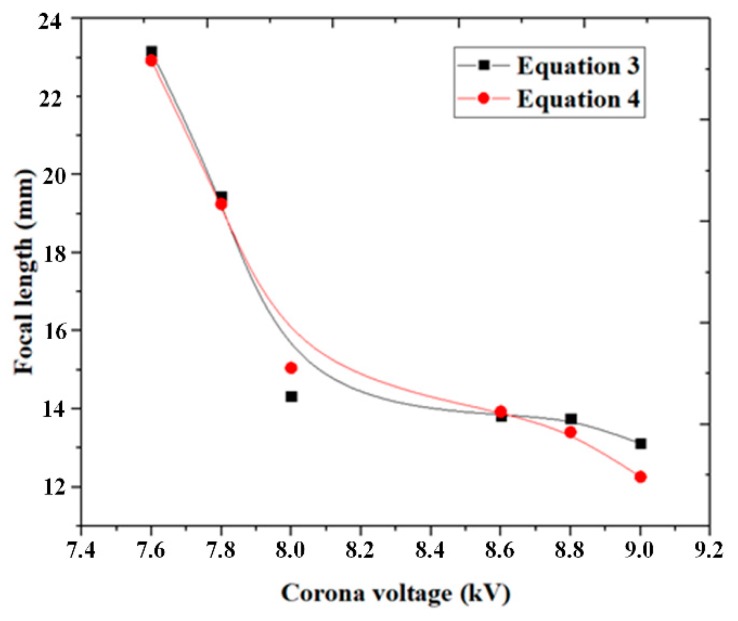
Focal length of the convex liquid lens as a function of the voltage.

Figure 8 shows the focal length of the liquid lens calculated by Equation (3) (black line with solid squares) and Equation (4) (red line with solid circles). The two solid lines were also obtained by fitting the calculated data according to the B-spline algorithm. The results from Equation (3) show that the focal length decreased from 22.9 to 12.2 mm with the increasing voltage, while the focal length ranged from 23.1 to 13.1 mm based on the results from Equation (4). The results from both equations are in good agreement, and the largest deviation of 7.3% occurred at a voltage of 9.0 kV. Therefore, the morphologies of the liquid lens were very close to a spherical shape.

**Figure 9:**
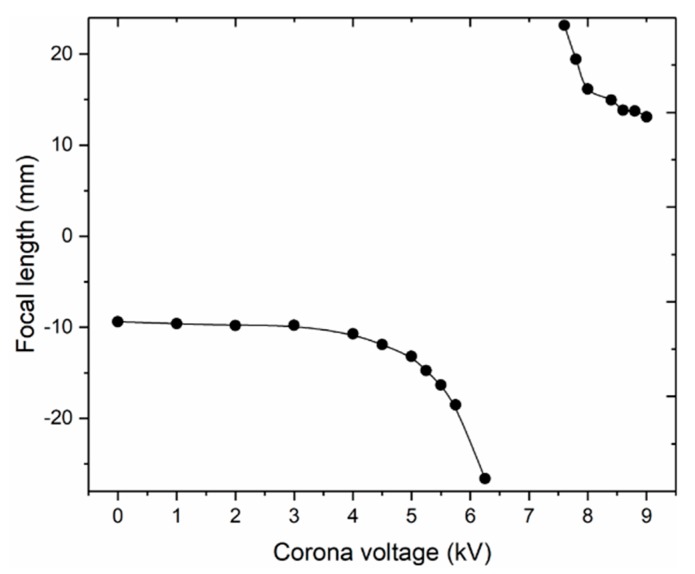
Focus range of the liquid lens with the voltage varying from 0 to 9.2 kV.

Figure 7 shows the detailed information of the images that we obtained using the Image J software. By comparing the images with and without the liquid lens, we could calculate the focal length of the liquid lens according to Equation (4). For example, we obtained the pixel length of the character “H” with and without the liquid lens and then measured the distance between the lens and the test object. From these measurements, we could get a focal-length value. If the images with and without the lens had a same-direction arrangement, the focal length was negative, which means that the lens interface was concave. When the images with and without the lens had an inverted arrangement, the focal length was positive, suggesting that the lens interface was convex. Based on this method, we could calculate the whole focus range of the liquid lens when the voltage was varied from 0 to 9.2 kV. Figure 9 presents the relationship between the focal length and the applied voltages. When the voltage increased from 0 to 6.25 kV, the focal length declined from −10 mm to negative infinity. When the voltage was further increased to about 7 kV, it turned into a convex lens and the focal length decreased from positive infinity to 10 mm.

**Figure 10:**
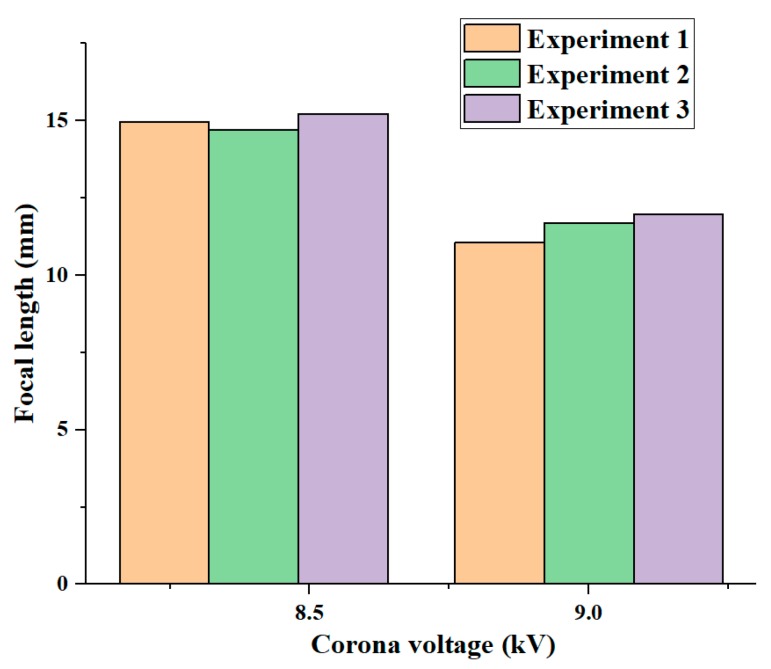
Focal length at two different voltages in three repeated experiments.

We conducted three experiments to test the robustness of this liquid-lens manipulation technique. The voltages of 8.4 kV and 9.0 kV were chosen, and at each voltage three experiments were repeated. Figure 10 shows three different experiments at voltages of 8.4 kV and 9.0 kV. At a voltage of 8.4 kV, the focal lengths were 14.9, 14.7, and 15.2 mm and their deviation was 0.5 mm. For a voltage of 9.0 kV, the focal lengths of the three experiments were, respectively, 11.1, 11.6, and 11.9 mm and their deviation was 0.8 mm.

**Figure 11:**
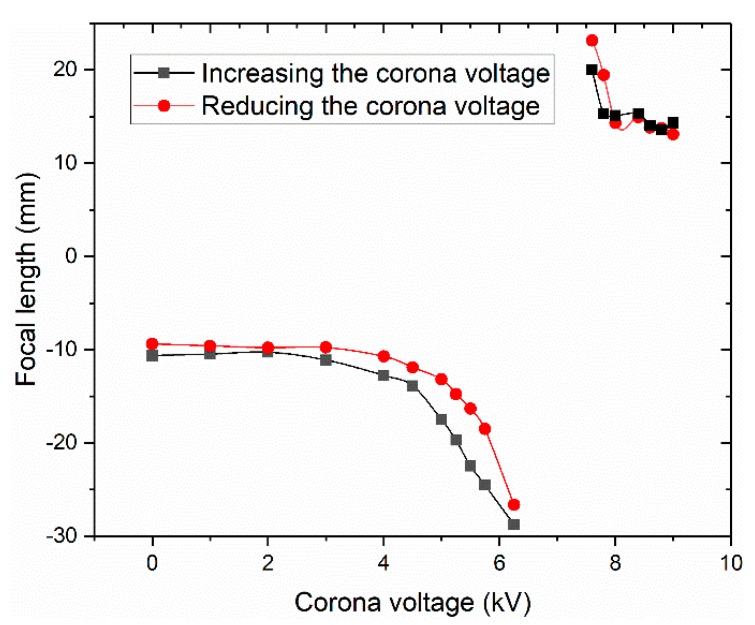
Focal length as a function of the voltage, which was gradually increased from 0 to 9.2 kV and then gradually decreased to 0 kV.

Figure 11 illustrates the focal length as a function of the voltage when we cyclically increased (solid squares) and decreased (solid circles) the voltage. A little hysteresis existed in the cycle. The majority of the focal lengths could be restored to their original state within a deviation of 2.5% after the corona voltage was increased from 0 to 9.2 kV and then returned to 0 kV.

## 4. Conclusions

In summary, a new method of realizing tunable-focus liquid lenses through charge injection was proposed and demonstrated. By a corona discharge in the air, electropressure with a magnitude of 10 Pa was generated at the interface between the liquid silicone and the air. Using only a 3D-printed U-tube and liquid silicone, the focus of the liquid lens varied from −∞ to −9 mm and from 13.11 mm to ∞. The robustness of this liquid-lens manipulation method was examined by testing the focus with an increasing–decreasing voltage loop. Such a simple and easily implemented liquid-lens manipulation method can be applied in many potential fields.
